# Retraction: In Vitro Study of Novel Collagenase (XIAFLEX®) on Dupuytren’s Disease Fibroblasts Displays Unique Drug Related Properties

**DOI:** 10.1371/journal.pone.0339148

**Published:** 2025-12-19

**Authors:** The PLOS One Editors

After this article [1] was published, concerns were raised regarding results presented in Figs 5, 9, 11, and S8.

Specifically,

- In Fig 5A the DD_Nodule Col-250ng and DD_Cord Col-250ng panels appear more similar than would be expected from independent results.
- In Fig 9, there appear to be similarities between the following panels despite representing different experimental samples:
  - Fig 9B DD_Nodule, Fig 9F DD_Skin, and Fig 9G DD_Nodule rotated 180°.
  - Fig 9F DD_Nodule bottom row, Fig 9F DD_Fat bottom row rotated 180°, and Fig 9G DD_Skin bottom row.
  - Fig 9F DD_Nodule top row and Fig 9G DD_Skin top row.
- The DD_Cord panels in Figs 9B, 9F and 9G appear similar, although they represent the same sample and experimental conditions.
- In Fig 9B when levels are adjusted to visualize the background, there appear to be areas where the background is discontinuous with the surrounding areas in the following panels:
  - DD_Nodule between the Untreated and Xia-400ng wells.
  - DD_Cord between the Untreated and Xia-400ng wells.
- In Fig 11A, part of the DD_Nodule suppWE panel appears similar to part of the DD_Skin suppWE panel.
- In Fig S8, there appear to be similarities between the following panels:
  - CT_Skin Xia-400ng and CT_Fascia Camp-250ng.
  - CT_Skin Col-250ng and CT_Fascia Xia-400ng.
  - CT_Skin Col-1000ng and CT_Fat Camp-250ng.
  - CT_Skin Camp-250ng and CT_Fascia Xia-700ng.
  - CT_Fat Xia-400ng and CT_Fat Xia-700ng.

Co-first author FS stated that in Fig 5A, comparable dot-plot distributions reflect biological reproducibility and shared gating templates. Regarding the panel similarities in Fig 9, they stated that in in-cell western blotting, well images appear alike when cell type, confluence, and treatment are similar. Regarding the background of Fig 9B DD_Nodule and DD_Cord panels, co-first author FS stated the differences in background may reflect contrast adjustments made for montage clarity. They stated the data underlying the results in [1] are no longer available. As such, PLOS does not consider the above concerns can be resolved.

In light of the above unresolved concerns that question the reliability of the reported results, the *PLOS One* Editors retract this article.

FS, ANT and AB did not agree with the retraction and stand by the article’s findings. SS, VK and SGEH either did not respond directly or could not be reached.
